# Human leukocyte antigen-incompatible living donor kidney transplantation after desensitization: experience from a major transplant centre in Mexico

**DOI:** 10.3389/ti.2026.16365

**Published:** 2026-05-07

**Authors:** Hugo Leonardo Reynoso de la Torre, Enrique Rojas-Campos, José I. Cerrillos-Gutiérrez, Luis A. Evangelista-Carrillo, Adriana Banda-López, Moises Cruz-Landino, Laura E. Aguilar-Fletes, Luis G. González-Correa, Edith V. Alatorre-Moreno, Perla E. Simancas-Ruiz, Cindy B. Salazar López, Alejandra G. Miranda-Díaz, Sylvia Totsuka Sutto, Ernesto G. Cardona Muñoz, Mauricio Carvallo-Venegas, Salvador Mendoza-Cabrera, Claudia A. Mendoza Cerpa, Carolina Romo-Álvarez, Kevin Javier Arellano-Arteaga, Jorge Andrade-Sierra

**Affiliations:** 1 Department of Nephrology and Organ Transplant Unit, Specialties Hospital, National Western Medical Centre, Mexican Institute of Social Security, Guadalajara, Mexico; 2 Medical Research Unit in Renal Diseases, Specialties Hospital, National Western Medical Centre, Mexican Institute of Social Security, Jalisco, Mexico; 3 University of Guadalajara, University Health Sciences Center, Department of Physiology, Institute of Experimental and Clinical Therapeutics (INTEC), Guadalajara, Jalisco, Mexico; 4 Department of Pathology, Specialties Hospital, National Western Medical Centre, Mexican Institute of Social Security, Guadalajara, Mexico; 5 Department of Internal Medicine. Hospital Civil de Guadalajara “Dr. Juan I. Menchaca”, Guadalajara, Jalisco, Mexico

**Keywords:** antibody-mediated rejection, desensitization, donor-specific antibodies, highly sensitized recipients, HLA-incompatible transplantation

Dear editors,

Kidney transplant recipients (KTRs) sensitized against human leukocyte antigens (HLA) represent a major clinical challenge because multiple donor-specific antibodies (DSAs) often lead to positive crossmatches and a high risk of graft loss from antibody-mediated rejection (AMR) [1]. This challenge is worsened in Latin America as there are fewer kidney-exchange programs and DSA biobanks, reducing access to compatible donors. Consequently, our institution—one of the most active transplant centers in Latin America—expanded living-donor transplantation for HLA-incompatible pairs through individualized desensitization.

We performed a single-center retrospective cohort study of 65 highly sensitized living-donor KTRs (2018–2022) undergoing individualized desensitization with intravenous immunoglobulin (IVIG) alone (200 mg/kg/day ×3) or plasmapheresis (PP)-based therapy (three sessions plus IVIG and rituximab [RTX] 375 mg/m^2^, 1–2 doses) with a ≥12-month follow-up (Table 1). Crossmatch testing (flow cytometry and complement-dependent cytotoxicity [CDC]) was performed prior to desensitization in all patients. Crossmatch results were either positive or negative by flow cytometry or CDC. Patients with detectable donor-specific antibodies (DSA), regardless of crossmatch result, were considered at increased immunological risk and therefore underwent desensitization according to institutional criteria.

**TABLE 1:** Baseline characteristics, immunological profile, and outcomes according to desensitization strategy.

Variable	IVIg therapy (n = 20)	PP + IVIg + RTX (n = 45)	*P* value
Gender of recipients-male, n (%)	13 (65%)	25 (55.5%)	0.48
Age of recipients (years)	34.5 ± 9.5	33.5 ± 9.0	0.68
Age of donors (years)	37 ± 10	38 ± 12	0.75
Time on dialysis (years)	3.7 ± 2.9	3.4 ± 2.5	0.66
Serum creatinine at 12 months (mg/dL)	1.3 ± 0.5	1.6 ± 0.6	0.88
eGFR, mL/min/1.73 m^2^	69 ± 27	75.5 ± 28	0.42
Flow cytometry crossmatch (FCXM) Positive Negative	10 (50%) 10 (50%)	24 (53.3%) 20 (44.4%)	0.74
CDC crossmatch (§) Positive Negative	0 0	2 (4.5%) 0	--
DSA flowby - luminex (MFI ≥1,000), median (IQR) Class I Class II	0.0 (0.0–5039) 2,112 (250–2,899)	2,736 (0.0–8,076) 7,551 (636–11105)	0.11 0.18
Related living donor n (%) Unrelated living donor n (%)	17 (85%) 3 (15%)	30 (66.7%) 15 (33.3%)	0.13
Transplant number, n (%) First Second	17 (85%) 3 (15%)	16 (35.6%) 29 (64.4%)	0.001*
Histocompatibility n (%) 2 haplotype 1 haplotype None haplotype	2 (10%) 10 (50%) 8 (40%)	1 (2.2%) 21 (46.7%) 23 (51.1%)	0.332
AR incidence n (%)	3 (15%)	10 (22.2%)	0.502
Graft lost n (%)	1 (5%)	4 (8.9%)	0.59
Infectious events n (%)	7 (35%)	20 (44.4%)	0.34

*p = <0.05.

Data are presented as mean ± SD or median (IQR).

§ CDC crossmatch positivity occurred only in the PP + IVIG + RTX group.

All patients received standard maintenance immunosuppression (tacrolimus, mycophenolate mofetil [MMF], and prednisone), primarily antithymocyte globulin (ATG) induction, infection prophylaxis, and protocol and indication biopsies. Primary endpoints were acute rejection (AR) incidence and 12-month estimated glomerular filtration rate (eGFR; CKD-EPI); secondary endpoints included graft survival and infectious events.

AR occurred in 20% of patients (all AMR), with no differences between regimens; 12-month eGFR and graft survival were favorable and comparable. Infection-related mortality was low (6.2%). Infectious complications (predominantly urinary tract infections [UTIs]) were comparable between regimens; viral rates were low (cytomegalovirus [CMV] 1.5%, poliomavirus BK 6.2%). All variables with p < 0.05 in univariate analysis were entered into a multivariable logistic regression model using a forward stepwise approach. Graft survival was analyzed by Kaplan–Meier and log-rank tests; two-sided p < 0.05 was considered significant. Analyses used SPSS™ version 23.

High *de novo* or preformed DSAs, particularly class II, are typically associated with complement activation, microvascular inflammation, increased AMR, and worse outcomes [2, 3]. However, in our cohort, pre-transplant class II DSAs did not predict AR, graft loss, or reduced eGFR. Recipients of triple therapy had higher median class II DSA MFI (7,551), reflecting risk-based selection, yet outcomes were similar to those receiving IVIG alone, including patients with MFI >1,000. Notably, lower MFI values (<1,000) have also been linked to immunological risk [4], so patients treated with IVIG alone despite appearing lower risk remain vulnerable and require close monitoring and individualized management. Overall AR rates, 1-year graft function, and survival were favorable and comparable to other desensitization series, including contemporary meta-analytic evidence showing high 1-year graft survival after IVIg/plasmapheresis/rituximab desensitization [5] and single-center cohorts reporting AR incidences in the same range with acceptable longer-term graft survival [6]. These results also align with evidence that desensitization may offer survival comparable to, or better than, remaining on dialysis or awaiting a compatible deceased-donor transplant [7]. Complement-activating anti-HLA DSAs are associated with higher risk of allograft loss and AMR [3], but we could not determine whether preformed DSAs were complement fixing or were eliminated after desensitization. A notable proportion of patients underwent transplantation despite a positive crossmatch. Although desensitization is sometimes used for recipients with positive flow-cytometry or CDC crossmatches and/or very high pre-transplant DSA MFI, prior studies report higher AMR rates and poorer graft survival in such cases [1].

In our cohort, ∼50% of IVIG-only and 53.3% of triple-therapy patients had a positive crossmatch, predominantly by flow cytometry; only 3.1% had a positive CDC crossmatch and underwent triple therapy, achieving CDC negativization prior to transplantation. One developed AMR at 12 months, which was successfully treated with preserved graft function (creatinine 1.0 mg/dL), while the other died from pneumonia and sepsis with a functioning graft. We observed no significant outcome differences by crossmatch status, whether comparing positive versus negative or flow-cytometry versus CDC positivity. Desensitization practices are heterogeneous across centers [8, 9]. We assessed immunological risk using flow cytometry and CDC crossmatches and Luminex SAB donor-specific antibody testing before desensitization. At our center, regimen selection was clinician-driven, influenced by immunological risk and the decision of the nephrology committee. We defined a sensitized patient (high risk) as one with a positive or negative flow cytometry crossmatch, with donor-specific antibodies (DSA) greater than 1000 MFI, and a history of exposure to sensitizing risk factors [10], with triple therapy used preferentially for higher DSA MFI (4,000–5,000), introducing indication bias that limits causal comparisons. Nevertheless, outcomes were encouraging, since sensitized recipients, including those with lower MFI, remain at substantial risk of AMR [4]. Protocol biopsies at 3–6 months are a strength: no subclinical AR was detected and all AR episodes were diagnosed on indication. Post-transplant DSA profiles were not available, so we could not determine whether AR episodes were driven by *de novo* or preformed DSAs. Beyond MFI, donor–recipient HLA compatibility was also relevant: in our regression analysis, fewer shared haplotypes was the only factor independently associated with increased AR risk, consistent with evidence that greater histocompatibility reduces rejection. Although follow-up was limited to 12 months, graft survival did not differ between groups and was comparable to other reports with similar AMR rates [5, 6]. Graft loss occurred in five recipients (6.2%), primarily due to immunological causes, namely hyperacute rejection (n = 1), acute antibody-mediated rejection (n = 2), and mixed rejection (n = 1), with one additional case attributable to BK virus–associated nephropathy.

Finally, although desensitization-related immunosuppression may increase infection risk, overall infectious complications—predominantly UTIs—were similar between regimens and comparable to non-sensitized KTRs at our center [11] with infrequent viral infections (CMV and BK). Overall mortality was 6.2%, entirely due to infectious causes, with deaths resulting from pneumonia and sepsis (n = 3) and COVID-19 (n = 1), consistent with contemporary desensitization cohorts [5], and was higher among patients with DSAs to both HLA classes (p = 0.032), with no differences between regimens. Extended follow-up is warranted to better define late infectious risk. Although desensitization remains controversial regarding rejection risk and graft survival, recent consensus supports individualized strategies based on each patient’s immunological risk profile [9].

Limitations include the retrospective design and short follow-up. A lack of systematic post-transplant immunomonitoring limit analysis of DSA kinetics, distinction of *de novo* versus preformed DSAs, and assessment of long-term antibody-mediated injury and graft survival. Protocol biopsies at 3–6 months are a strength of the study—no subclinical AR was detected and all AR episodes were diagnosed on indication.

Overall, these findings support individualized desensitization as a feasible strategy to safely expand access to living-donor kidney transplantation in Latin America.

## Data Availability

The raw data supporting the conclusions of this article will be made available by the authors, without undue reservation.
